# P112: Hand hygiene practice in new zealand hospitals one year into the hand hygiene New Zealand programme

**DOI:** 10.1186/2047-2994-2-S1-P112

**Published:** 2013-06-20

**Authors:** JT Freeman, Christine Sieczkowski, Louise Dawson, Hayley Callard, Andrew Keenan, Sally Roberts

**Affiliations:** 1 Auckland District Health Board, Auckland, New Zealand

## Introduction

Hand Hygiene New Zealand (HHNZ) is a nationalquality improvement programme funded since June 2011 by the Health Quality and Safety Commission (HQ & SC). The goal ofHHNZ is to use the World Health Organization (WHO) 5 Moments model to improve hand hygiene compliance nationally. During its first year, HHNZ has benefitted enormously from support provided by the Hand Hygiene Australia (HHA) programme. Benefits have included auditor training sessions in NZ led by HHA auditors, use of the HHA Smartphone auditing application and the HHAcomputerised data management system.

## Methods

For the four monthly audit period ending 31 October 2012, all 20 District Health Boards (DHBs) in NZ submitted data. The total number of moments audited was 29128 and correct hand hygiene was performed on 18095/29128 occasions, giving an overall compliance rate of 62.1% (range: 61.6%-62.7%). When examined by healthcare worker category, medical practitioners had the lowest rate (57.1%) and phlebotomists the highest (72.5%). When examined by “Moment”; higher rates were observed for “after” moments than “before” moments (“before patient contact” 56.6% versus “after patient contact” 71.1%; and “before a procedure” 55.7% versus “after a procedure or body fluid exposure risk” 67.2%).

## Conclusion

These results indicate that although there is still much work to be done, the HHNZ programme is gaining traction in NZ hospitals at the end of its first year. Important strategies for the coming year include launching educational initiatives specificallytargeting senior doctors and medical opinion leaders. Such initiatives will focus on “*when* and *why*” hand hygiene is necessaryto ensure patient safety. Finally, collaboration with HHA during the last year has been hugely beneficial for the HHNZ programme andprovides a potential model for future international collaborations.

## Disclosure of interest

None declared

